# Yeast V-ATPase Proteolipid Ring Acts as a Large-conductance Transmembrane Protein Pore

**DOI:** 10.1038/srep24774

**Published:** 2016-04-21

**Authors:** Sergio Couoh-Cardel, Yi-Ching Hsueh, Stephan Wilkens, Liviu Movileanu

**Affiliations:** 1 Department of Biochemistry & Molecular Biology, SUNY Upstate Medical University, Syracuse, New York 13210, USA; 2 Department of Physics, Syracuse University, 201 Physics Bldg., Syracuse, New York 13244-1130, USA; 3 Structural Biology, Biochemistry, and Biophysics Program, Syracuse University, 111 College Place, Syracuse, New York 13244-4100, USA; 4 The Syracuse Biomaterials Institute, Syracuse University, 318 Bowne Hall, Syracuse, New York 13244-1200, USA

## Abstract

The vacuolar H^+^ -ATPase (V-ATPase) is a rotary motor enzyme that acidifies intracellular organelles and the extracellular milieu in some tissues. Besides its canonical proton-pumping function, V-ATPase’s membrane sector, V_o_, has been implicated in non-canonical functions including membrane fusion and neurotransmitter release. Here, we report purification and biophysical characterization of yeast V-ATPase *c* subunit ring (*c*-ring) using electron microscopy and single-molecule electrophysiology. We find that yeast *c*-ring forms dimers mediated by the *c* subunits’ cytoplasmic loops. Electrophysiology measurements of the *c*-ring reconstituted into a planar lipid bilayer revealed a large unitary conductance of ~8.3 nS. Thus, the data support a role of V-ATPase *c*-ring in membrane fusion and neuronal communication.

The vacuolar H^+^ -ATPase (V-ATPase; V_1_V_o_-ATPase) is a large multisubunit enzyme complex responsible for ATP hydrolysis-driven acidification of subcellular compartments in all eukaryotic organisms and the extracellular space in some tissues^1^,^2^,^3^. V-ATPase’s proton-pumping function is vital for basic cellular processes, including pH homeostasis, endocytosis, protein trafficking, signaling, neurotransmitter release, hormone secretion, bone remodeling, sperm maturation, and urine acidification. The V-ATPase from *S. cerevisiae*, a well-characterized model system for the enzyme from higher organisms, is composed of 14 different polypeptides with varying stoichiometries and can be divided into a V_1_ catalytic sector (A_3_B_3_(C)DE_3_FG_3_H), and a membrane-integral V_o_ proton translocating sector (*ac*_8_*c*’*c*”*de*) (Fig. 1)^4^,^5^,^6^. Eukaryotic V-ATPase is a rotary motor enzyme related to F- and A-ATP synthase found in bacteria, mitochondria and archaea^7^,^8^. In the V-ATPase, ATP hydrolysis on V_1_ is coupled to proton-pumping across V_o_ *via* rotation of a central stalk made of V_1_ and V_o_ subunits DF*c*_8_*c*’*c*”*d*. A peripheral stator composed of V_1_ subunits E, G, H, and C serves to stabilize the motor by binding to the N-terminal domain of the V_o_ *a* subunit (*a*_NT_).

Protons are translocated across the membrane by the rotating ring of *c* subunits (“proteolipids”), with each *c* subunit carrying a proton binding carboxyl group between two aqueous half channels located at the interface of the membrane-integral C-terminal domain of subunit *a* (*a*_CT_) and the *c* subunit ring (*c*-ring). The yeast V-ATPase *c*-ring is composed of three isoforms *c, c*’ and *c*” in the ratio 8:1:1^6^,^9^, with *c* and *c*’ having each four and *c*” five transmembrane α helices. However, unlike the related F- and A-ATPases, eukaryotic V-ATPase is regulated *in vivo* by a unique mechanism referred to as “reversible dissociation”, a condition under which the enzyme disassembles into membrane-bound V_o_ and cytoplasmic V_1_ sectors in a nutrient-dependent fashion^10^,^11^. Upon disassembly, V_1_ no longer hydrolyzes MgATP^12^ and free V_o_ does not catalyze passive proton translocation^13^. Reversible dissociation of V-ATPase is well characterized in *S. cerevisiae*^14^, but more recent studies suggest that the mammalian enzyme is regulated by a similar process in some cell types^15^,^16^,^17^,^18^,^19^.

Besides its canonical proton-pumping function in the holo V-ATPase, the free V_o_ or parts thereof, have been implicated in membrane fusion and neurotransmitter release^20^,^21^,^22^,^23^,^24^,^25^,^26^ (reviewed in references^27^,^28^). For example, V_o_^20^, and specifically *a*_NT_^21^,^22^,^23^,^24^, have been shown to participate in *trans*-SNARE pairing in a calcium- and calmodulin-dependent manner^26^, and it has been speculated that these V_o_-SNARE interactions may assist in vacuole fusion in yeast^21^ or spontaneous neurotransmitter release^26^. Other studies have suggested a direct role of the V_o_ *c*-ring in fusion and/or neurotransmitter release by either catalyzing lipid mixing in the late stage of synaptic vesicle fusion with the presynaptic membrane^21^,^25^, or by allowing efflux of neurotransmitters through its central pore^21^. However, due to contradictory reports^29^, possibly caused by compensatory pathways *in vivo*, and the lack of a well-defined *in vitro* system, the physiological significance of the proposed non-canonical functions of the V_o_ have not been firmly established.

Here, we describe purification as well as structural and functional characterization of yeast V-ATPase *c*-ring. We report that purified *c*-ring forms dimers *via* an interaction of the *c* subunits’ cytoplasmic loops. Using single-molecule electrophysiology measurements, we further show that the *c*-ring functions as a transmembrane protein pore with a large unitary conductance of ~8.3 nS, a finding that is in accord with the *c*-ring’s pore diameter of ~3.5 nm, as estimated from cryo-electron microscopy (cryo-EM) images of *c*-ring two-dimensional (2-D) crystals and homology modeling. Taken together, this work provides the first direct evidence that eukaryotic V-ATPase *c*-ring exhibits some of the properties required for membrane fusion and neurotransmitter release.

## Results

### Purification and Structural Characterization of Yeast V-ATPase *c*-ring

**Figure 2:**
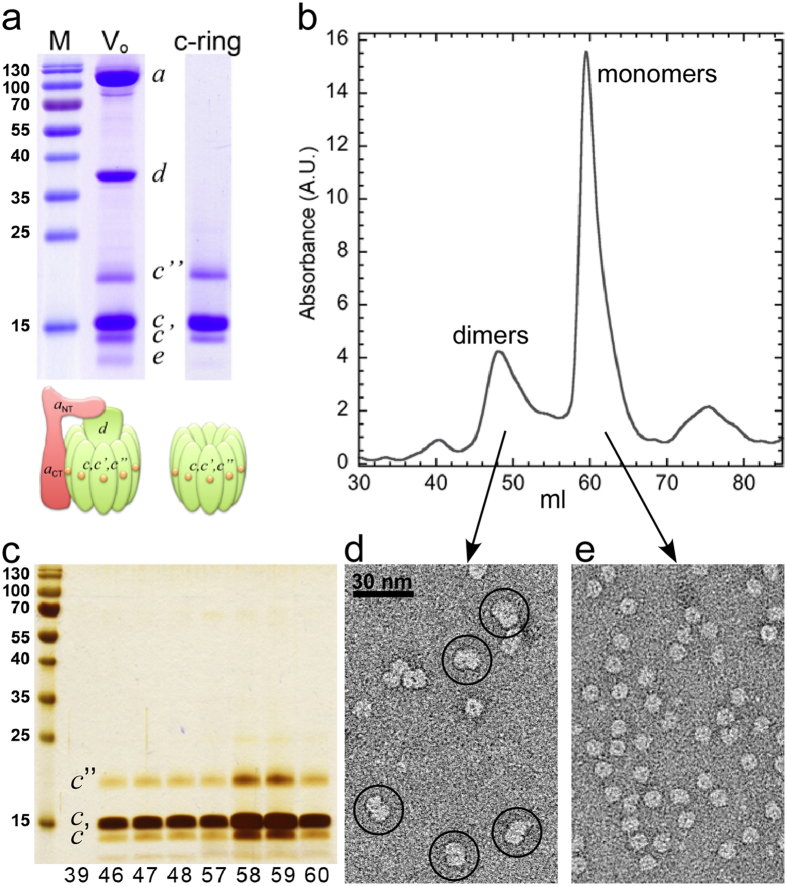
Purification and structural characterization of yeast V-ATPase *c*-ring. (**a**) SDS-PAGE of purified V_o_ (10 μg) as well as dialyzed and concentrated *c*-ring (5 μg). The gels were stained with Coomassie blue. (**b**) Size-exclusion chromatography (Superdex 200, 16 × 500 mm^2^) of *c*-ring (1 mg) in 0.1% DDM containing buffer. (**c**) Peak fractions (10 μl each) were analyzed by SDS-PAGE and silver staining. (**d,e**) Negative stain EM of fraction 48 and 58, respectively. Dimeric *c*-ring complexes are highlighted by circles in (**d**).

We recently developed a protocol for isolating milligram quantities of yeast V-ATPase V_o_ membrane sector and found that treatment of V_o_ with the ionic detergent lysophosphatidyl glycerol (LPPG) leads to a selective release of subunit *d* from the complex to form the V_o_Δ*d* (*ac*_8_*c*’*c*”*e*) subcomplex^30^. Here, we show that by adding a second ionic detergent, sodium lauroyl sarcosinate (SLS) followed by ammonium sulfate precipitation, both *d* and *a* subunits are removed, thus producing a pure yeast V-ATPase *c*-ring. Following ammonium sulfate precipitation, *c*-ring was extensively dialyzed against detergent (e.g. dodecyl maltoside (DDM)) containing buffer to remove LPPG and SLS. The dialyzed sample (up to 200 ml depending on the initial amount of V_o_) was then concentrated to ~1–2 ml for further purification. Figure 2a shows SDS-PAGE of purified yeast V_o_ and *c*-ring after dialysis and concentrating. As can be seen in Fig. 2a, the *c*-ring sample showed proteolipid bands (*c, c*’ and *c*”) with a staining ratio as seen in intact V_o_, suggesting that *c*-ring stayed intact during the procedure. Following dialysis, concentrated *c*-ring was subjected to size-exclusion chromatography on Superdex 200 for further purification and for removal of excess detergent accumulated from concentrating the large volume after dialysis (Fig. 2b). SDS-PAGE and silver staining revealed that the first two peaks seen in the elution profile contained *c*-ring (Fig. 2c), whereas the third peak contained only very little protein and was probably due to small-molecule contaminations.

To analyze the oligomeric state of the *c*-rings eluted from the column, we visualized fractions 48 and 58 by negative-stain EM. As can be seen, fraction 48 showed predominantly projections of dumbbell-shaped molecules consistent with *c*-ring dimers (Fig. 2d), whereas fraction 58 contained ring-shaped molecules with a stain-filled central cavity (Fig. 2e). The appearance of monomeric and dimeric *c*-rings in the two fractions is consistent with the apparent molecular sizes of the two peaks, which were ~110 and ~370 kDa, respectively (the calculated molecular mass of *c*_8_*c*’*c*” is ~170 kDa). We also purified *c*-ring using undecyl and decyl maltoside (UnDM and DM), either all the way from the extraction stage through gel filtration, or by extracting with DDM and exchanging detergent to UnDM or DM on the gel-filtration column. Under all these conditions, both dimers and monomers of the *c*-ring were obtained, albeit in different ratios (Supplementary Fig. S1).

**Figure 3:**
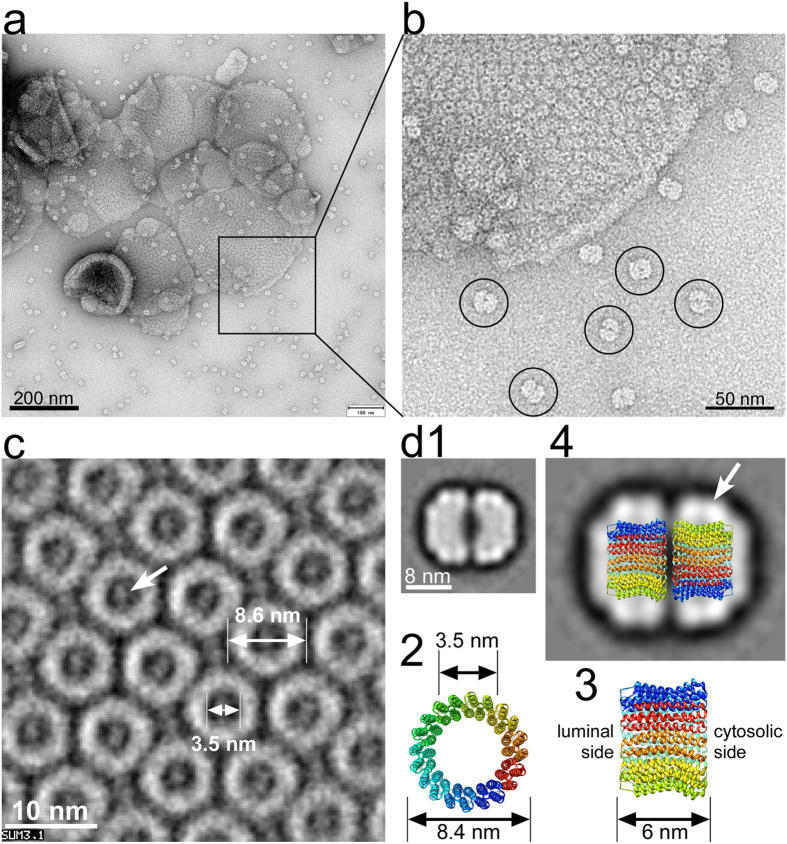
2-D crystallization and single-particle image analysis of *c*-ring. For 2-D crystallization, *c*-ring (1.5 mg/ml) was mixed with DOPC (0.5 mg/ml) and detergent was removed by the addition of BioBeads. (**a**) Negative stain TEM of DOPC reconstituted *c*-ring after detergent removal for 7 days. At this stage, para-crystalline arrays of *c*-rings were visible, but reconstitution was incomplete with numerous dimeric *c*-ring complexes visible next to lipid vesicles (see molecules highlighted by circles in (**b**)). (**c**) Reconstituted *c*-rings after complete detergent removal were imaged by cryo-EM and crystalline areas were analyzed by correlation averaging. (**d1**) Averaged projection (367 images) of dimeric *c*-ring. A dataset of 4337 images of dimeric *c*-rings from images, as shown in (**b**) was analyzed by reference free alignment and classification, as implemented in EMAN1.9. (d2,3) Top- and side-view of a bacterial V-ATPase *c*-ring (K_10_ ring from *E. hirae*; 2bl2.pdb)^31^. (d4) Model of the dimeric yeast V-ATPase *c*-ring superimposed on the EM average. The stain-excluding detergent belt is indicated by the arrow.

We recently reported conditions under which purified yeast V_o_ forms 2-D crystals in DOPC bilayers^30^. Reconstituting *c*-ring under the same conditions and visualizing resulting lipid vesicles before complete detergent removal revealed bilayers with closely packed *c*-rings next to dumbbell-shaped molecules outside the bilayers (Fig. 3a,b) reminiscent of the images of dimeric *c*-ring observed in fraction 48 after gel filtration (Fig. 2d). Cryo-EM of the *c*-ring containing liposomes, vitrified after complete detergent removal (21 days), revealed small crystalline areas suitable for structure analysis by correlation averaging. The averaged projection image of one of these crystalline patches shows closely packed ring-shaped molecules with outer and inner diameters of ~8.6 and 3.5 nm, respectively (Fig. 3c), very similar to the dimensions of the homo-decameric *c*-ring of the bacterial sodium-pumping V-ATPase from *E. hirae* (K_10_; 2bl2.pdb)^31^ (Fig. 3d2). Interestingly, some density appears to occupy the central pore of the *c*-rings (see arrow in Fig. 3c). The nature of this density is unclear at this time, but it is possible that the ‘plug’ is formed by the N-terminal α helix of the single copy subunit *c*” isoform and/or lipid molecules, as has been described for the *c*-rings isolated from F-ATPase^32^. We next generated a dataset of 4337 of the dumbbell-shaped molecules highlighted in Fig. 3b, which was analyzed by reference free alignment protocols and sorted into 16 classes. Figure 3d1 shows a class average (367 images) of the predominant side-view projection of the dimeric molecules, which we interpret to be composed of two *c*-rings bound to each other *via* their cytoplasmic loops (Fig. 3d4) and with stain-excluding detergent belts covering the hydrophobic exterior of each individual ring (see arrow in Fig. 3d4). Taken together, the data show that pure *c*-ring can be obtained from V-ATPase V_o_ and that, in the absence of the *a* and *d* subunits, the *c*-ring dimerizes *via* the *c* subunit cytoplasmic loops.

### Single-molecule Electrophysiology of the *c*-ring

**Figure 4:**
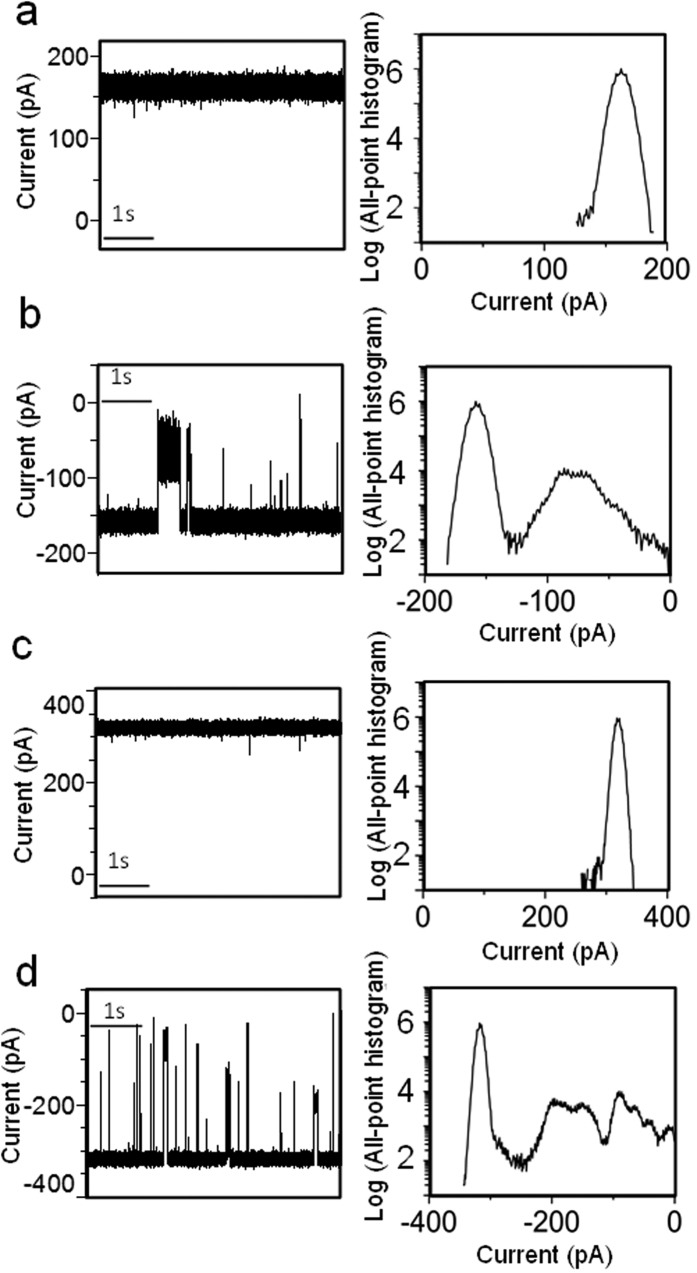
Representative single-channel electrical traces along with their corresponding semi-logarithmic all-point, current-amplitude histograms. The traces were acquired with the *c*-ring at various applied transmembrane potentials. (**a**) +20 mV. (**b**) −20 mV. (**c**) +40 mV. (**d**) −40 mV. All single-channel traces were low-pass Bessel filtered at a frequency of 10 kHz. These electrical traces are typical among n = 8 distinct single-channel electrical recordings.

We used single-channel electrical recordings^33^ to examine the electrophysiological features of yeast V-ATPase *c*-ring (see Supplementary Fig. S2 for a schematic of the electrophysiology setup). Remarkably, the *c*-ring formed uniform, large-conductance transmembrane pores with a single-channel conductance of 8.33 ± 0.24 nS when reconstituted in planar lipid bilayers, and consistent with an ohmic ion conduction through the pore lumen (n = 66; Supplementary Fig. S3). The *c*-ring always inserted into a planar lipid membrane in the same orientation, as judged by the asymmetry of the gating activity depending on the voltage polarity. Representative single-channel traces, which were acquired at potentials between −50 and +50 mV, are presented in Fig. 4. The single-channel electrical traces acquired at positive potentials (Fig. 4a,c) revealed a fairly quiet signature, exhibiting brief and infrequent current spikes. On the contrary, at negative transmembrane potentials (Fig. 4b,d), the *c*-ring showed a dynamic voltage-induced gating activity, encompassing a broad range of current blockades with amplitudes between 10 and 100% of that corresponding to the unitary conductance. The duration of current blockades spanned a time interval of several orders of magnitude, between sub-millisecond values and tens of seconds (Supplementary Fig. S4). The overall frequency of these transient current blockades was ~1.7 s^−1^ and ~4.8 s^−1^ at a transmembrane potential of −20 and −40 mV, respectively. Using a logarithmic likelihood ratio (LLR) test for comparing different fitting models of these voltage-induced current blockades with a confidence number C = 0.95^34^, we determined that the closed single-channel current fluctuations fall into three-exponential probability distributions (Supplementary Figs S5 and S6). Fits to four-exponential probability distribution models were not significantly better, as judged by the LLR value. The open-state events or the inter-current blockade intervals underwent a single exponential, indicating that, at negative applied potentials, the *c*-ring showed a current gating dynamics with one open sub-state and three closed sub-states. Large negative transmembrane potentials, greater than −30 mV, frequently produced either irreversible or reversible current blockades to the fully closed sub-state (Supplementary Fig. S7).

### Electrophysiology of the *c*-ring - Subunit *d* Interaction and Conductance of holo V_o_

**Figure 5:**
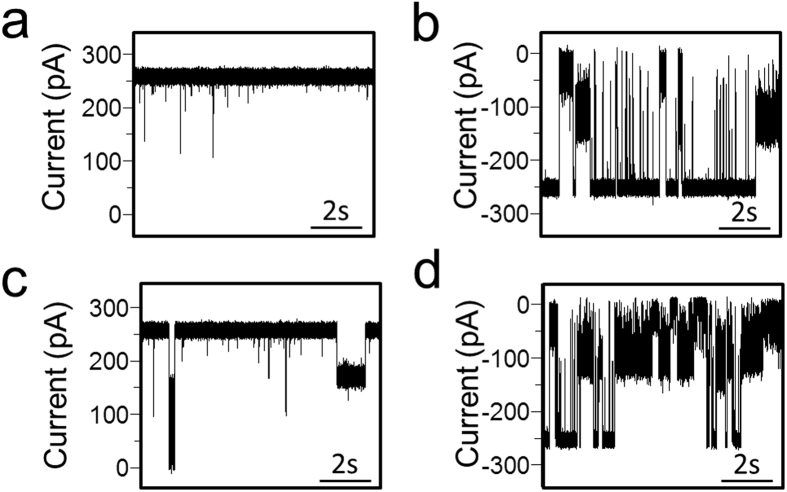
Interaction of the *d* subunit with the *c*-ring produces current blockades of varying amplitude. In panels (**a,b**) single-channel electrical signature of the *c*-ring is illustrated at +30 and −30 mV, respectively. In (**c,d**) the single-channel traces are (**a,b**) in the presence of 0.3 μM *d* subunit, respectively, which was added to the *cis* side. These electrical traces are typical among n = 3 distinct single-channel electrical recordings.

When the negatively charged *d* subunit was added to the *cis* side (corresponding to the cytosolic side), the open-state conductance was decorated by infrequent, long-lived single-channel events in the range of seconds. In Fig. 5, we illustrate typical single-channel electrical traces of the *c*-ring at transmembrane potentials of +30 and −30 mV, as well as in the absence (Fig. 5a,b) and presence of 0.3 μM *d* subunit (Fig. 5c,d). It is likely that these long-lived current blockades are produced by individual binding events between the negatively charged *d* subunit and the positively charged cytosolic domains of the *c* subunits. A much more intense and complex gating activity was observed at negative transmembrane potentials, which culminated with the irreversible full closure of the *c*-ring when 0.45 μM *d* subunit was added to the *cis* side of the chamber (Supplementary Fig. S8).

**Figure 6:**
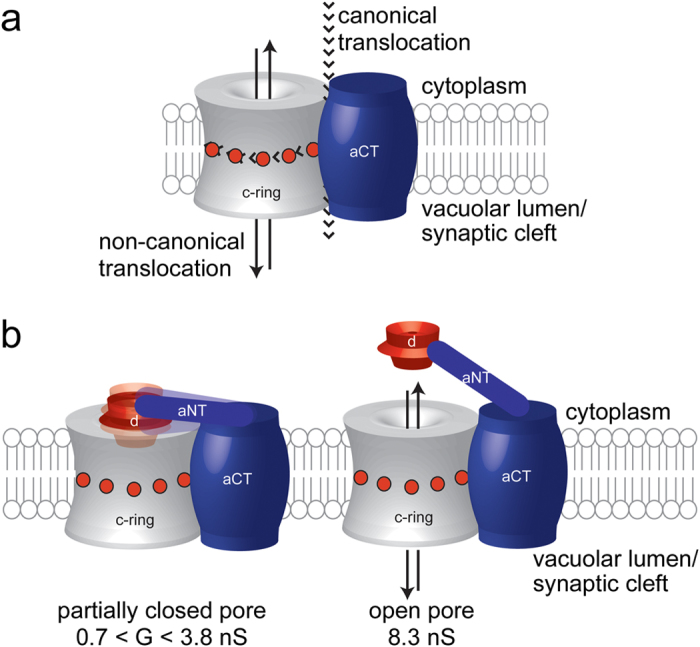
Canonical and non-canonical ion translocation across the Vo complex/*c*-ring. (**a**) Schematic model of the *a*_CT_-*c*-ring complex highlighting canonical and non-canonical ion translocation pathways. (**b**) Dispersity in the unitary conductance of the V_o_ complex was likely caused by structural fluctuations or different conformations of the *a*_NT_-*d* complex and its interaction with the *c*-ring cytoplasmic domains.

Finally, we were able to record individual single-channel insertions of the entire V_o_ complex into a planar lipid bilayer. In contrast to the results obtained with the *c*-ring, we noticed a broader distribution of the unitary conductance with an average of 1.81 ± 0.84 nS (n = 47) and within the range between 0.7 and 3.8 nS (Supplementary Fig. S9). A disparity among the unitary conductance values of V_o_ recorded in this work suggests different conformations of subunit *d* and/or *a*_NT_ with respect to the *c*-ring central pore within V_o_ (Fig. 6). In addition, we occasionally detected unitary conductance values greater than those in this range, in six distinct situations, when the applied potential was between +30 and +100 mV. This finding suggests that in these instances, the *a*_NT_-*d* subcomplex dissociated completely from the opening of the *c*-ring.

## Discussion

Here, we show that treating purified yeast V-ATPase V_o_^30^ with LPPG and SLS followed by ammonium sulfate precipitation allowed isolation of pure and intact yeast V-ATPase *c*-ring. SDS-PAGE of the *c*-ring preparation confirmed the presence of all three proteolipid isoforms (*c, c*’, and *c*”) with relative staining intensities of the bands as seen in V_o_ preparations, indicating that the *c*-ring complex is not damaged during the purification procedure. Furthermore, the similar staining intensity of the *c*’ and *c*” bands is consistent with the reported 1:1 stoichiometry of the two isoforms^9^. Structural integrity of the *c*-ring was also confirmed by gel filtration and EM analysis of single particles and 2-D crystals. While the resolution of the projection images obtained from 2-D crystals was insufficient to determine the subunit stoichiometry, the outer and inner diameters of the rings are consistent with the size of the related *E. hirae* K_10_ ring^31^, and the *c*-ring as seen in the ~7 Å cryo-EM map of holo yeast V-ATPase^6^. Interestingly, from gel filtration experiments together with single-particle EM, we found that *c*-ring exists in a monomer-dimer equilibrium, with the dimer interface likely formed by the cytoplasmic loops of the *c* subunits.

Such a dimeric arrangement had been proposed earlier from studies with proteolipids isolated from arthropod hepatopancreas (referred to as “ductin”^35^,^36^) or from presynaptic membranes isolated from the *Torpedo* electric organ (referred to as “mediatophore”^28^,^37^). Primary sequence analysis of ductin and mediatophore polypeptides revealed virtual identity to the V-ATPase *c* subunit^38^,^39^,^40^, and in one instance it was shown that expression of arthropod ductin could complement the *c* subunit deletion (vma3Δ) phenotype (Vma^−^) in yeast^41^. From these studies, it was speculated that dimeric ductin and mediatophore oligomeric structures could function in cell-cell communication including neurotransmitter release^26^,^28^,^42^,^43^,^44^. It should be noted that the 2-D crystal projection images of yeast *c*-ring presented here appear very similar to images of ductin hexagonal arrays even though the ductin rings were modeled to contain only six *c* subunit monomers^36^. From subsequent studies in yeast and *Drosophila*, it was speculated that dimerization (*trans*-complex formation) of V-ATPase *c*-rings could catalyze membrane fusion events *in vivo*^21^,^25^ and that V_o_’s *a*_NT_ could change conformation and serve as a SNARE like protein to assist in fusion of synaptic vesicles with the presynaptic membrane^23^. However, subsequent experiments in yeast challenged some of these proposals on the basis that it is V-ATPase-driven acidification and not V_o_ *per se* that is required for the fusion process^29^. In summary, while the observed tendency of the V-ATPase *c*-rings to form stable dimers appears to lend support to above proposals, it remains to be seen whether the *c*-ring dimerization occurs *in vivo* and if so, what its physiological function may be.

In this work, single-molecule electrophysiology examinations revealed a large-conductance of the *c*-ring, with a rich gating activity at negative, but not positive potentials. This non-canonical ion conductance through the pore lumen of the *c*-ring is not to be confused with the transmembrane pathway of the canonical proton-pumping function of V_o_ in holo V-ATPase, which occurs at the interface of *a*_CT_ and *c*-ring (Fig. 6a). The asymmetric single-channel electrical signature depended on the polarity of the applied potential, which indicated that the *c*-ring consistently inserted into the planar lipid bilayer with a preferred orientation. This directionality of insertion is likely due to the charge distribution in the *c* subunits. At physiological pH, the cytoplasmic domains of the *c, c*’ and *c*” subunits carry +3, +4, and +1 net charges, respectively, for a total of 29 positive charges (Supplementary Fig. S10). We reason that under conditions of positive potential in *trans*, the *c*-ring insertion always occurred with the luminal side first, leaving the positively charged cytoplasmic domains oriented towards the negative *cis* side of the chamber. Therefore, a positive potential in our case meant a positive potential in the vacuolar lumen side of the *c*-ring with respect to its cytosolic side. This configuration was chosen for our setup, because it corresponds to the physiological situation where the cytoplasmic side is negative relative to the vacuolar lumen (or outside of the cell). This is in accord with measured membrane potentials across plant or yeast vacuolar membranes, which are between +30 to +40 mV^45^,^46^. The observed current fluctuations in situations where the polarity was switched to negative in the *trans* side after insertion could then be explained by electrostatic forces either pulling the positively charged cytoplasmic domains of the *c* subunits or pushing their negatively charged C-termini in the vacuolar lumen towards the pore interior. Both types of structural changes would likely lead to the frequent and long-lived current blockades, which were observed to encompass a broad range of amplitudes and durations. Another possibility is that the N-terminal α helix of *c*” is transiently changing conformation depending on the polarity of the applied membrane potential, with stronger or more frequent fluctuations under conditions of negative potential. Moreover, at potentials below −40 mV with respect to the cytosolic side, we frequently observed irreversible current blockades to a low-amplitude current state, suggesting that the polarity of the potential is important for the biological function of *c*-ring.

Recently, it was reported that the *c*-ring of the related F_1_F_o_-ATP synthase possesses voltage-sensitive, large-conductance properties^47^. The persistent opening of the F_o_ *c*-ring led to a rapid depolarization of the inner mitochondrial membrane, suggesting that this transmembrane pathway is a candidate for the mitochondrial permeability transition pore. This raises the possibility that the large-conductance properties as well as the associated non-canonical functions of the proteolipid rings are conserved among rotary ATPase membrane sectors.

As mentioned above, a positive potential in our setup meant a positive potential on the lumenal side of the *c*-ring relative to the cytosolic side, as is observed *in vivo*^45^,^46^. To study the interaction between the *c*-ring and the capping *d* subunit, as monitored by the change in *c*-ring conductance, both components were added to the *cis* side of the chamber at a positive potential. Under these conditions, we observed that the conductance of the *c*-ring decreased from ~8.3 nS to lower values (0.7 < G < 3.8 nS), consistent with a specific interaction between the capping *d* subunit and the cytoplasmic domains of the *c*-ring, resulting in a partial and reversible current blockage of the pore. Subunit *d* is highly negatively charged (−23 at pH 7, −26 at pH 8), suggesting that its interaction with the positively charged cytoplasmic domains of the *c*-ring is at least partly driven by electrostatic forces. It is conceivable that the polarity of the applied potential alters the exposure of these cytoplasmic *c*-ring domains with respect to the membrane surface. At positive potentials, for example, *c*-ring’s positively charged domains are oriented towards the pore exterior. On the contrary, at negative potentials the electrostatic forces pull the positively charged cytoplasmic domains towards the pore lumen. In this way, their voltage-driven structural alterations likely affect the binding interactions with the capping *d* subunit.

Unlike the *c*-ring, which showed a uniform conductance of ~8.3 nS, holo V_o_ exhibited a range of unitary conductance values, with an average value smaller than that observed for the *c*-ring. This finding suggests that *a*_NT_ and subunit *d* may exist in different conformations that block the central pore of the *c*-ring to various degrees. Very rarely, in less than 10% of the successful V_o_ insertions, and especially under conditions of higher applied potentials (e.g., +100 mV), a unitary conductance of ~8 nS was observed, suggesting complete (but reversible) release of the *a*_NT_-*d* complex from the cytoplasmic opening of the *c*-ring as illustrated in Fig. 6b. This outcome can be rationalized with the structure of the intact V_o_. Recently, we obtained a 3-D EM reconstruction of detergent-solubilized V_o_ sector that showed *a*_NT_ in a close contact with the *d* subunit^30^, an interaction not seen in the reconstructions of holo V_1_V_o_^6^. Using recombinant V-ATPase subunits, we found that *a*_NT_ binds to subunit *d* with a moderate affinity of ~5 μM^30^. While the affinity between *c*-ring and subunit *d* has not been measured, there is evidence that the interaction is not very tight. For example, *in vivo* experiments showed that shortening of the tether connecting *a*_NT_ and *a*_CT_ resulted in partly assembled membrane sectors (*ac*_8_*c*’*c*”) that did not have subunit *d* bound, unless *d* was significantly over-expressed^48^. Furthermore, 3-D reconstructions of lipid nanodisc-reconstituted V_o_, calculated at slightly higher resolution than our recent reconstruction of detergent-solubilized protein^30^, revealed that the *d* subunit is pushed deeper into the central pore of the *c*-ring upon binding of V_1_ to V_o_ (Stam *et al.*, *in preparation*). Taken together, this means that subunit *d* is bound to V_o_ *via* two low-affinity interactions that, on one hand, result in a high-avidity binding of *d* to V_o_, but at the same time allow for structural changes that are required for the (re)assembly of the holo V-ATPase. On the other hand, release of *a*_NT_ from its interaction with *d* in free V_o_, likely accompanied by pore opening, is also consistent with the proposed role of *a*_NT_ as a SNARE binding partner^26^.

Interestingly, from electrophysiology studies with yeast vacuolar vesicles reconstituted into planar lipid bilayers, it was reported that vacuolar membranes contain a calcium-dependent ion channel with a conductance of 0.43 nS (at 0.3 M KCl)^49^. While it is known that yeast vacuolar membranes contain a significant fraction of free V_o_ at any given time^14^, it remains to be seen whether the conductance observed in vacuolar membrane vesicles is in any way related to the conductance of free V_o_ observed in our study. One question is how does the single-channel conductance of the *c*-ring relate to the inner dimensions of the pore lumen? Are the here reported single-channel currents in accord with the diameter of the fully open pore? From cryo-EM projections of the *c*-ring 2-D crystals, we estimated an internal diameter of ~3.5 nm (Fig. 3c). However, homology modeling reveals that the internal surface of the pore is corrugated and that the internal diameter varies from ~3.1 nm on the vacuolar side to ~3.9 nm on the cytoplasmic side (Supplementary Fig. S11). The pore length, as measured by the side chain-to-side chain distance is ~6.5 nm. The unitary conductance, *G*, should be the reciprocal of the pore resistance. However, the measured resistance should also include the access resistance of the pore^50^. If we consider a highly simplified model of a cylindrical pore, the equation of its unitary conductance is the following^51^,^52^:


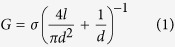


where σ is the bulk conductivity of ionic solution, whereas *d* and *l* are the diameter and length of the pore, respectively. In this case, *σ* is 10.5 S m^−1^ for 1 M KCl at 23 °C^53^. The relatively large internal size of the pore, as compared with conventional ion channels, and high salt concentration of the buffer solution justify for neglecting the effects of the surface charges on the single-channel conductance. Under these contexts, for a cylindrical geometry with a length of ~6.5 nm, the single-channel conductance is ~8.9 and ~10.9 nS for a diameter of ~3.1 and ~3.5 nm, respectively. It is worth mentioning that in the structure of the *c*-ring it is possible that a ‘plug,’ which is formed by the N-terminal α helix of the single copy subunit *c*” isoform, partitions into the pore lumen, thus reducing its single-channel conductance. On the other hand, using equation (1), we can estimate of the average internal diameter of the pore for a given single-channel conductance. For *G* = 8.3 nS and *l* = 6.5 nm, we obtain *d* = 3.0 nm, which is quite close to the constricted region of the pore. These simplified estimates suggest that the measured single-channel conductance of the *c*-ring is in accord with the diameter of the fully open pore. Nevertheless, we are aware that such estimates neglect other effects and forces, such as the internal bilayer pressure and surface tension of the membrane, which might alter the overall cross-sectional geometry and internal pore diameter.

### Conclusion and Future Prospects

Our structural and electrophysiological characterization of the *c*-ring provides support for the proposed non-canonical functions of the V-ATPase membrane sector. The observed dimerization is consistent with the proposed *trans*-complex formation of the *c*-ring that precedes membrane fusion by lipid mixing, whereas the large conductance of the pore could explain the proposed role of the *c*-ring in neurotransmitter release. However, while more work will be needed to establish the physiological role of the here reported properties of the *c*-ring, the stability and unique conductance properties of the pore could lead to novel applications in emerging arenas of nanobiotechnology, molecular biomedical diagnosis, as well as therapeutics. Experiments towards these aims are ongoing in our laboratories.

## Materials and Methods

### Reagents

Dodecyl-, undecyl- and decyl-β-D-maltopyranoside (DDM, UnDM and DM) were from Anatrace and 1-palmitoyl-2-hydroxy-sn-glycero-3-[phospho-RAC-(1-glycerol)] (LPPG) was obtained Avanti. Sodium lauroyl sarcosinate (SLS) was from Sigma. Cadmodulin beads were obtained from GE Healthcare or Agilent. All other reagents were of analytical grade from Sigma-Aldrich.

### Purification of yeast V-ATPase V_o_ sector

All steps were carried out at 4 °C unless noted otherwise. Yeast V-ATPase V_o_ was purified *via* affinity chromatography as previously described^30^ with the following modifications. The protein A moiety that is part of the TAP tag was eliminated from the chromosomal DNA of the yeast strain used for V_o_ isolation (YSC1178-7502926; Open Biosystems) by homologous recombination. At the same time, the *his3* marker used to introduce the TAP tag in the commercial TAP strain was replaced with *ura3*. Briefly, cells were resuspended in 25 mM Tris-HCl, pH 7.5, 500 mM sorbitol, 2 mM EDTA (Tris-sorbitol) and a protease inhibitor cocktail was added before mechanical disruption with a bead beater (Omni MES) in a 960 ml Mason jar. Release of arginine as measured with a commercial kit (Megazyme) was used as a measure of vacuole breakage and for optimizing lysis conditions. Unbroken cells and cell walls were removed by low-speed centrifugation (900 × g, 10 min) and the supernatant was centrifuged at 16,000 × g for 30 minutes to pellet mitochondria and other organelles. Total microsomes were then pelleted at 265,000 × g for 2 hours and washed once in Tris-sorbitol. Membrane pellets were resuspended in Tris-sorbitol and after measuring the protein concentration, membranes were stored at −80 °C until use. For V_o_ purification, membranes were extracted after adding protease inhibitors using DDM or UnDM (0.6 mg of detergent per mg of protein). Buffers to wash and elute the column contained two times the critical micelle concentration (CMC) of DDM (0.02%) or UnDM (0.06%) unless noted otherwise.

### Purification of V-ATPase *c*-ring

Isolation of yeast V-ATPase *c*-ring was adapted from reference^54^ with the following modifications. Purified yeast V_o_ diluted to 0.5–1 mg/ml using 10 mM Tris-HCl, pH 8, 10 mM β-mercaptoethanol (BME), 0.5 mM EGTA, and 0.02% DDM or 0.06% UnDM (CaM elution buffer) was incubated with 1% SLS and 0.05% LPPG for one hour at 42 °C. Ammonium sulfate was then added to 65% saturation and incubated for 4 hours at room temperature. Precipitated subunits *a* and *d* were removed by centrifugation at 14,000 × g for 30 minutes at room temperature and the supernatant was filtered through a 0.45 μm membrane. The filtered sample was then extensively dialyzed (3 × 2 l over 2 days) against CaM elution buffer in a 50 kDa cutoff dialysis bag at room temperature. The final dialysate (100–300 ml) was concentrated to ~1–2 mg/ml *c*-ring in a 50 kDa cutoff Vivaspin concentrator at 4 °C. At this stage, the yield of *c*-ring was on average 0.4 mg per mg of V_o_, corresponding to a recovery of about 75%. Between 0.5–2 mg of *c*-ring was applied to a Sephadex S200 (16 × 500 mm^2^) column attached to an AKTA FPLC system and eluted in CaM elution buffer using a flow of 0.6 ml/min. Fractions were analyzed by 13% SDS-PAGE gel electrophoresis. Gels were stained with Coomassie Blue or silver. Peak fractions for *c*-ring dimer and monomer were concentrated in 50 kDa cutoff Vivaspin concentrators.

### Two dimensional (2D) crystallization

2D-crystals of V-ATPase *c*-ring were generated using 1.5 mg/ml *c*-ring and 0.45 mg/ml DOPC, as described for intact V_o_^30^.

### Electron Microscopy

For negative staining, 5 μl of samples (*c*-ring dimers or monomers or *c*-ring 2D-crystals) were applied to glow-discharged carbon-coated copper grids and stained with 1% uranyl acetate or uranyl formate. If necessary, samples were diluted with CaM elution buffer containing 0.02% DDM to prevent *c*-ring aggregation. For cryo-EM, 3 μl of 2-D crystallization mixtures were applied to holey carbon coated copper grids (C-flat 400 mesh, 2/2) and vitrified in liquid ethane in a home built plunger. Grids were examined in a JEM-2100 (JEOL) transmission electron microscope operating at 120 or 200 kV. Images were recorded in minimum dose mode using a charge-coupled device (TVIPS F415MP) at 40,000× optical magnification and an underfocus of 1.5 μm. Catalase was used as calibration standard.

### Image Analysis

Single-particle analysis of a dataset of 4,337 projections of *c*-ring dimers was done with the EMAN1.9 package of programs^55^ using the reference free alignment script *refine2d.py*. The dataset was sorted into 16 classes and since *c*-ring dimer was almost exclusively oriented on the carbon support to produce the dumbbell-shaped side view projection, essentially all the class averages had a very similar appearance. Analysis of 2-D crystalline *c*-ring patches was done with the IMAGIC 5 package of programs^56^ using correlation averaging as previously described^57^.

### Single-channel electrical recordings

Protein reconstitution into planar lipid bilayers was probed by electrical recordings^58^,^59^. The two sides of the chamber, *cis* and *trans* (1.5 ml each), were separated by a 25 μm-thick Teflon septum (Goodfellow Corporation). An aperture in the septum, ~80 μm in diameter, was pretreated with hexadecane (Sigma-Aldrich), which was dissolved in highly purified pentane (Fisher) at a concentration of 10% (v/v). Both the *cis* and *trans* chambers contained 1 M KCl, 10 mM Tris, pH 8.0. A planar lipid bilayer of 1,2 diphytanoyl-sn-glycero-phosphatidylcholine (Avanti) was formed across the aperture. Monomeric *c*-ring (extracted and purified in UnDM and containing less than 1% detergent) was added to the *cis* chamber to a final concentration of ~0.2–0.8 ng/ml. Single-channel currents were acquired by using an Axopatch 200B patch-clamp amplifier (Axon) in the whole-cell mode (β = 1) with a CV-203BU headstage. The *cis* chamber was grounded, meaning that a positive current represents positive charge moving from the *trans* to the *cis* side. A Precision T3500 Tower Workstation Desktop PC (Dell) was equipped with a DigiData 1322 A A/D converter (Axon) for data acquisition. Single-channel electrical traces were low-pass filtered with an 8-pole Bessel filter (Model 900; Frequency Devices) at a frequency of 10 kHz and sampled at 50 kHz. pClamp 10.3 software (Axon) was used for data acquisition and analysis.

### Other Procedures

Protein concentration was measured using the bicinchoninic acid (BCA) method (Thermo Scientific) in combination with TCA precipitation, as described in reference^60^ using fatty acid free BSA as standard. Recombinant yeast V-ATPase subunit *d* was purified as previously described^30^. Native gel electrophoresis (BN- and hrCN-PAGE) was performed according to reference^61^.

## Additional Information

**How to cite this article**: Couoh-Cardel, S. *et al.* Yeast V-ATPase Proteolipid Ring Acts as a Large-Conductance Transmembrane Protein Pore. *Sci. Rep.* **6**, 24774; doi: 10.1038/srep24774 (2016).

## Supplementary Material

Supplementary Information

## Figures and Tables

**Figure 1:**
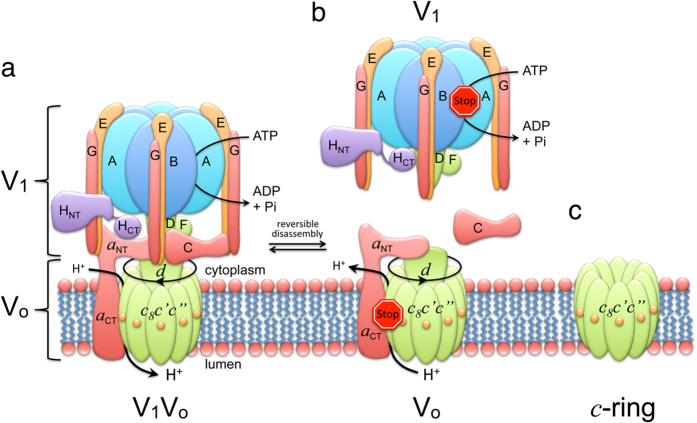
Subunit architecture and regulation of yeast V-ATPase. (**a**) Schematic of yeast V-ATPase subunit architecture. Rotor subunits (DF*dc*_8_*c*’*c*”) are in green, the stator complex ((EG)_3_CH*a*) is in orange and the catalytic hexamer (A_3_B_3_) is in blue (adapted from reference^30^). Yeast V-ATPase *c* subunit ring (*c*-ring) contains three proteolipid isoforms, *c, c*’ and *c*”. While *c* and *c*’ each contain four, *c*” is predicted to contain five transmembrane segments with the N-terminal α helix that is not found in *c* and *c*’. This α helix is likely located inside the central pore of the *c*-ring^62^,^63^. While *c*” is conserved across species, the *c*’ isoform has as of yet not been identified in the mammalian enzyme. (**b**) Regulation of V-ATPase’s ATP hydrolysis-driven proton pumping activity by reversible dissociation into inactive V_1_ and V_o_ sectors. (**c**) Yeast V-ATPase *c*-ring.
