# Supplementary material for: Yeast V-ATPase Proteolipid Ring Acts as a Large-conductance Transmembrane Protein Pore
Source: Sci Rep. 2016 Apr 21;6:24774. doi: 10.1038/srep24774 (PMC4838861; doi:10.1038/srep24774)
Supplement: Supplementary Information [file srep24774-s1.pdf]

Supplementary Information

Yeast V-ATPase Proteolipid Ring Acts as a Large-conductance Transmembrane Protein Pore

Sergio Couoh-Cardel,[1]* Yi-Ching Hsueh,[2]* Stephan Wilkens[1] and Liviu Movileanu[2,3,4]

[1]Department of Biochemistry & Molecular Biology, SUNY Upstate Medical University, Syracuse, New York 13210, USA

[2]Department of Physics, Syracuse University, 201 Physics Bldg., Syracuse, New York 13244-1130, USA

[3]Structural Biology, Biochemistry, and Biophysics Program, Syracuse University, 111 College Place, Syracuse, New York 13244-4100, USA

[4]The Syracuse Biomaterials Institute, Syracuse University, 318 Bowne Hall, Syracuse, New York 13244-1200, USA

Short Title: Channel Features and Dimerization of Yeast V-ATPase Proteolipid Ring
Keywords: Biochemistry; Biophysics & Structural Biology; Vacuolar ATPase; membrane transport; proteolipid ring; electrophysiology; membrane fusion; proton pump; protein structure; electron microscopy

*These two authors contributed equally to this work.

Correspondence: Liviu Movileanu, Ph.D., Department of Physics, Syracuse University, 201 Physics Bldg., Syracuse, New York 13244-1130, USA. Phone: 315-443-8078; Fax: 315-443-9103; E-mail: lmovilea@syr.edu; Web: http://movileanulab.syr.edu/

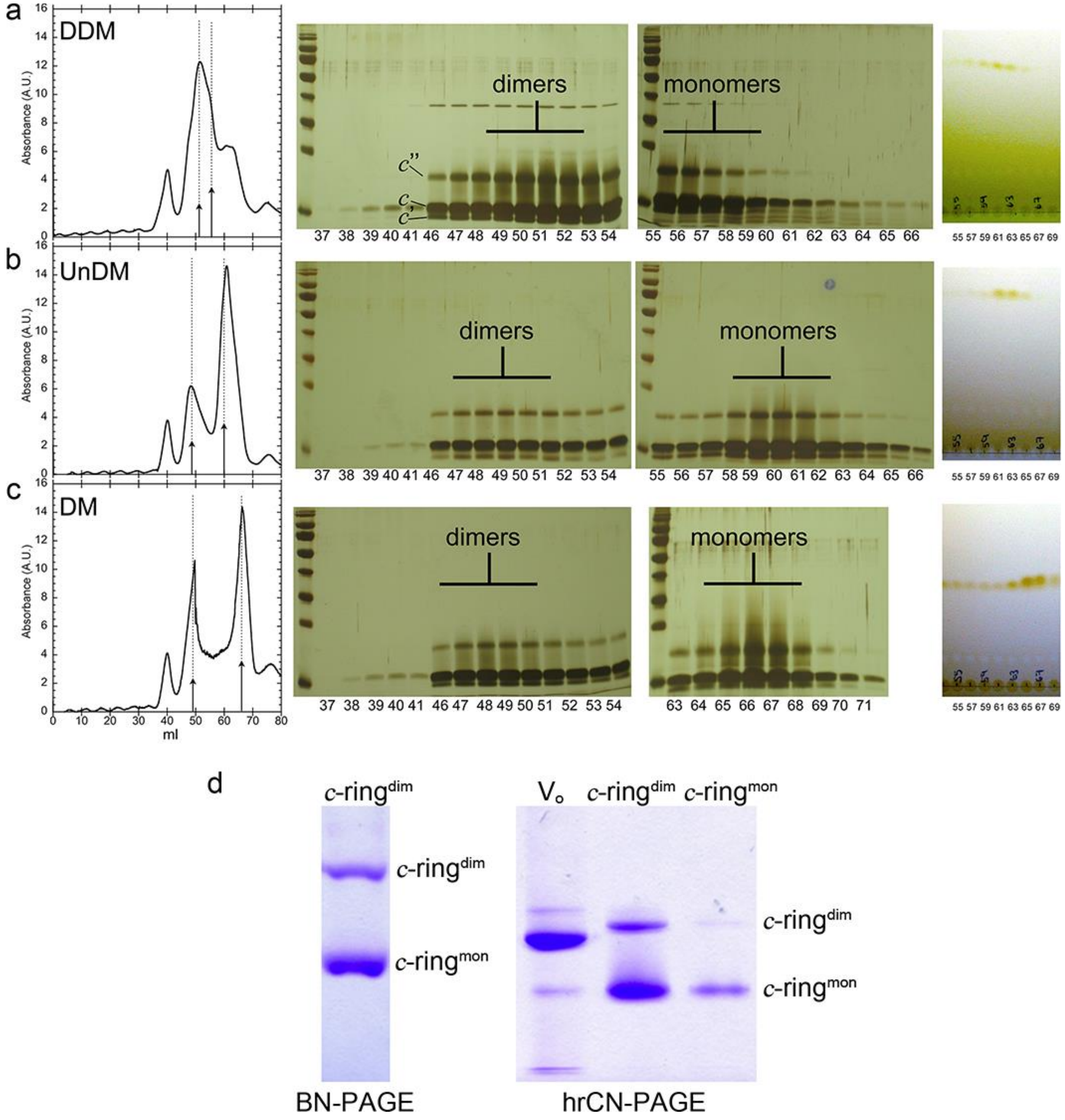

Figure S1: Size-exclusion chromatography of yeast V-ATPase *c*-ring in dodecyl, undecyl and decyl maltoside. Yeast total membranes were extracted with 0.6 mg DDM per mg of membrane protein and V_o was purified using 0.1% DDM in all buffers. Following removal of subunits *a* and *d* with LPPG/sarkosyl and ammonium sulfate precipitation, *c*-ring was dialyzed against 0.1% DDM containing buffer. *c*-ring was concentrated to ~2.7 mg/ml. ~2 mg was subjected to size-exclusion chromatography in either 0.02% DDM (**a**), 0.06% UnDM (**b**) or 0.18% DM (**c**). Fractions were analyzed by 13% SDS-PAGE and silver staining. The detergent content of the fractions was analyzed using thin-layer chromatography in chloroform/methanol/water (100:38.5:6.2 v/v) and visualized with iodine vapor. (**d**) Analysis of *c*-ring using blue native (BN) and high-resolution clear native (hrCN) 3-11% gradient PAGE[1]. Left gel: 5 µg dimeric *c*-ring purified in UnDM. The negatively charged Coomassie Blue dye and/or the electric field leads to partial dissociation of the *c*-ring dimers. Right gel: 5 µg V_o, 5 µg of dimeric, and ~1 µg of monomeric *c*-ring were analyzed.

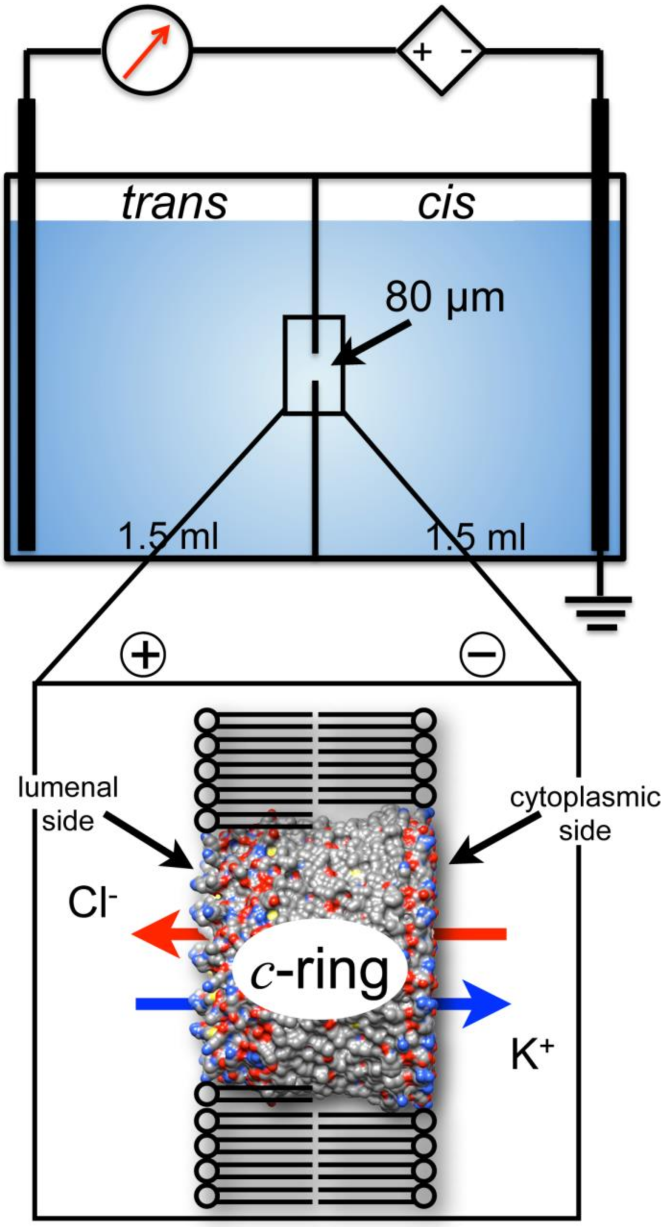

Figure S2: Schematic of the single-molecule electrophysiology setup. Both the *cis* and *trans* sides of the chamber contained each 1.5 ml of 10 mM Tris-HCl, pH 8.0, 1 M KCl. The chambers were separated by a planar lipid bilayer of 1,2 diphytanoyl-sn-glycero-phosphatidylcholine, which was formed across a teflon aperture with a diameter of ~80 µm. Monomeric *c*-ring, which was extracted and purified in UnDM and containing less than 1% detergent, was added to the *cis* side to a final concentration of ~0.2-0.8 ng/ml. The *cis* side was grounded, meaning that a positive current represents positive charge moving from the *trans* to the *cis* side, as depicted in the schematic. Electrophysiology measurements were conducted as described in references [2,3].

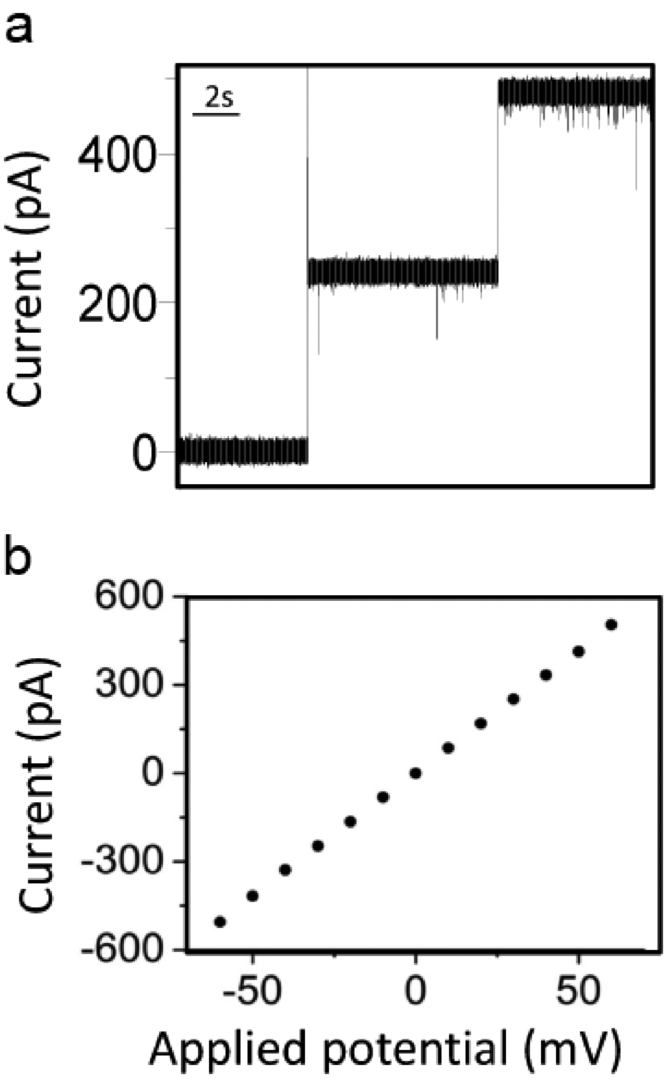

Figure S3: The c-ring transmembrane protein pore of the V-type ATPase shows a uniform large-conductance signature. (**a**) Two consecutive single-channel insertions of the c-ring monomer recorded at a transmembrane potential of +30 mV, resulting in 242 pA and 483 pA current levels. This electrical recording illustrates the uniformity of the single-channel conductance of the *c*-ring transmembrane protein pore with a value of ~8 nS; (**b**) The current-voltage profile is represented as average over at least three independent single-channel experimental determinations. This profile reveals a linear relationship with a slope of ~8.3 nS, which is consistent with an average conductance of 8.33 ± 0.24 nS (n=66), as derived from different single-channel determinations at different applied transmembrane potentials.

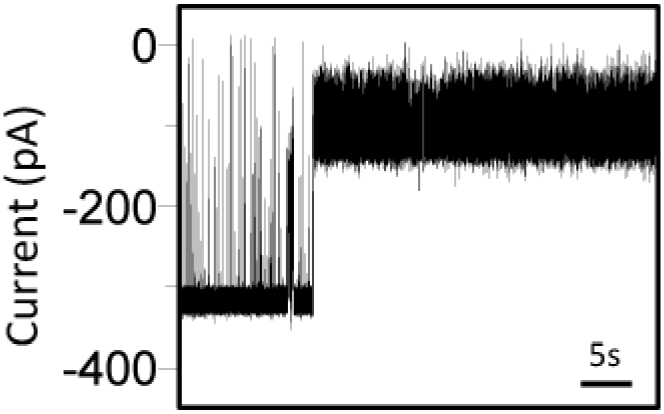

Figure S4: A representative long-lived current blockade with a duration of ~50 s, which was recorded at a transmembrane potential of -40mV. These long-lived current blockades were either reversible or irreversible. They were not detected at transmembrane potentials of -20 mV or great than this value (e.g., -10 mV).

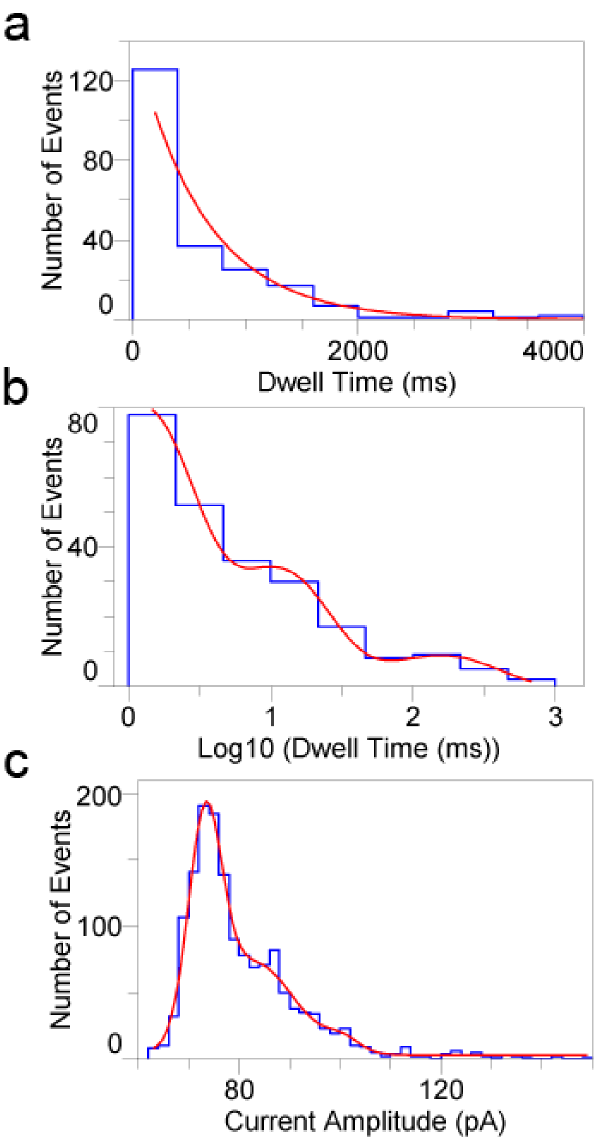

Figure S5: Dwell time and current amplitude histograms derived from a typical single-channel electrical recording acquired with the *c*-ring transmembrane protein pore at an applied transmembrane potential of -20 mV. (**a**) A representative dwell-time histogram of the open events. τ_O = 616 ± 93 ms. For the fitting approach, we used a logarithmic likelihood ratio (LLR) test with a confidence number C=0.95[4,5]. The fitting method was variable metric on the exponential probability function[6]. χ^2_{crit} = 5.99, $\chi^2_{1\rightarrow2}$ = -1179; (**b**) A representative dwell-time histogram of the closed events. The fitting of this histogram indicated three time constants, τ_{C1} = 1.09 ± 0.04 ms, τ_{C2} = 10.1 ± 0.06 ms, and τ_{C3} = 156.8 ± 0.17 ms, with the normalized probabilities P_{C1} = 0.63 ±0.02, P_{C2} = 0.29 ± 0.01, and P_{C3} = 0.08 ± 0.01, respectively. The fitting method was variable metric on the exponential logarithmic-probability function for revealing time constants spanning over a three-order of magnitude range. We used a LLR test with a confidence number C=0.95. The correlation coefficient was R = 0.963. χ^2_{crit} = 5.99, $\chi^2_{1\rightarrow2}$=354.8, $\chi^2_{2\rightarrow3}$=37.90, $\chi^2_{3\rightarrow4}$=0.14; (**c**) A representative current-amplitude histogram fitted with a three-component Gaussian, revealing current blockade peaks of I_{B1} = 73 ± 1 pA, I_{B2} = 83 ± 1 pA, and I_{B3} = 100 ± 2 pA with the normalized probabilities of P_{B1} = 0.46 ± 0.05, P_{B2} = 0.51 ± 0.09, and P_{B3} = 0.03 ± 0.02, respectively. The events list file was generated from a single-channel electrical trace with a duration of 135.7 s. pClamp 10.5 software (Axon Instruments) was used for data analysis after the single-channel traces were low-pass Bessel filtered at a frequency of 5 kHz.

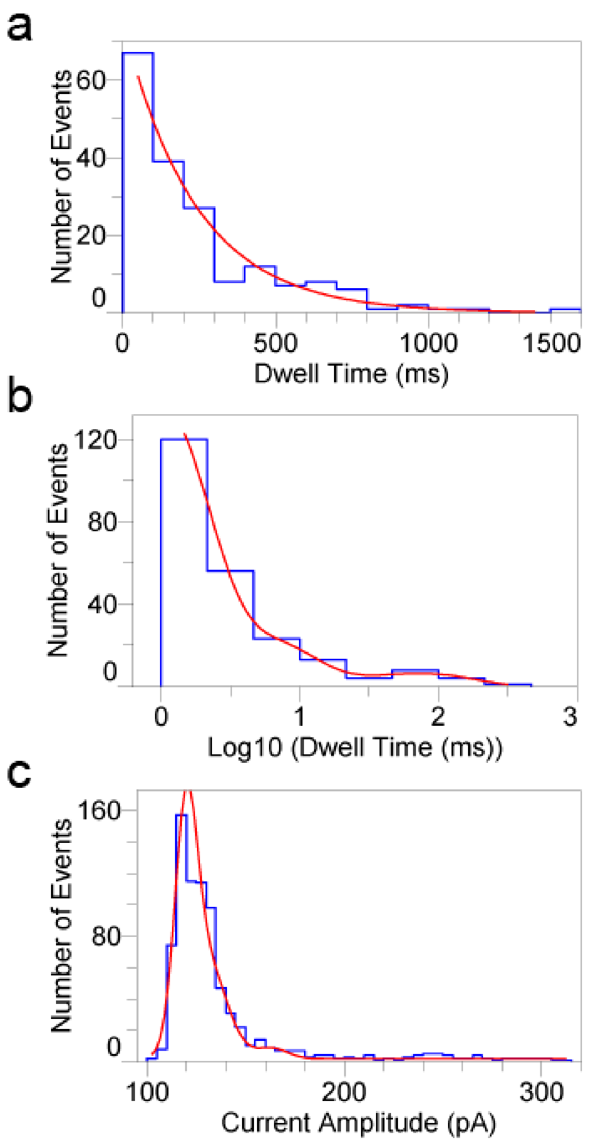

Figure S6: Dwell time and current amplitude histograms derived from a typical single-channel electrical recording acquired with the c-ring transmembrane protein pore at an applied transmembrane potential of -40 mV. (**a**) A representative dwell time histogram of the open events. $\tau_O = 238 \pm 20$ ms. For the fitting approach, we used a logarithmic likelihood ratio (LLR) test with a confidence number C=0.95[4,5]. The fitting method was variable metric on the exponential probability function[6]. $\chi^2_{crit} = 5.99$, $\chi^2_{1\rightarrow2} = 1.7$; (**b**) A representative dwell-time histogram of the closed events. The fitting of this histogram indicated three time constants, $\tau_{C1} = 0.847 \pm 0.057$ ms, $\tau_{C2} = 4.80 \pm 0.16$ ms, and $\tau_{C3} = 71.20 \pm 0.31$ ms. The normalized probabilities were $P_{C1} = 0.82 \pm 0.03$, $P_{C2} = 0.14 \pm 0.02$, and $P_{C3} = 0.04 \pm 0.01$, respectively. The fitting method was variable metric on the exponential logarithmic-probability function for revealing time constants spanning over a three-order of magnitude range. We used a LLR test with a confidence number C=0.95. The correlation coefficient was R = 0.964. $\chi^2_{crit} = 5.99$, $\chi^2_{1\rightarrow2} = 360.5$, $\chi^2_{2\rightarrow3} = 14.6$, $\chi^2_{3\rightarrow4} = -1.8\times10^{-5}$; (**c**) A representative current-amplitude histogram fitted with a three-component Gaussian, revealing current blockade peaks of $I_{B1} = 116 \pm 1$ pA, $I_{B2} = 127 \pm 1$ pA, and $I_{B3} = 146 \pm 7$ pA with the normalized probabilities of $P_{B1} = 0.31 \pm 0.01$, $P_{B2} = 0.50 \pm 0.08$, and $P_{B3} = 0.19 \pm 0.07$, respectively. The events list file was generated from a single-channel electrical trace with a duration of 47.7 s. pClamp 10.5 software (Axon Instruments) was used for data analysis after the single-channel traces were low-pass Bessel filtered at a frequency of 5 kHz.

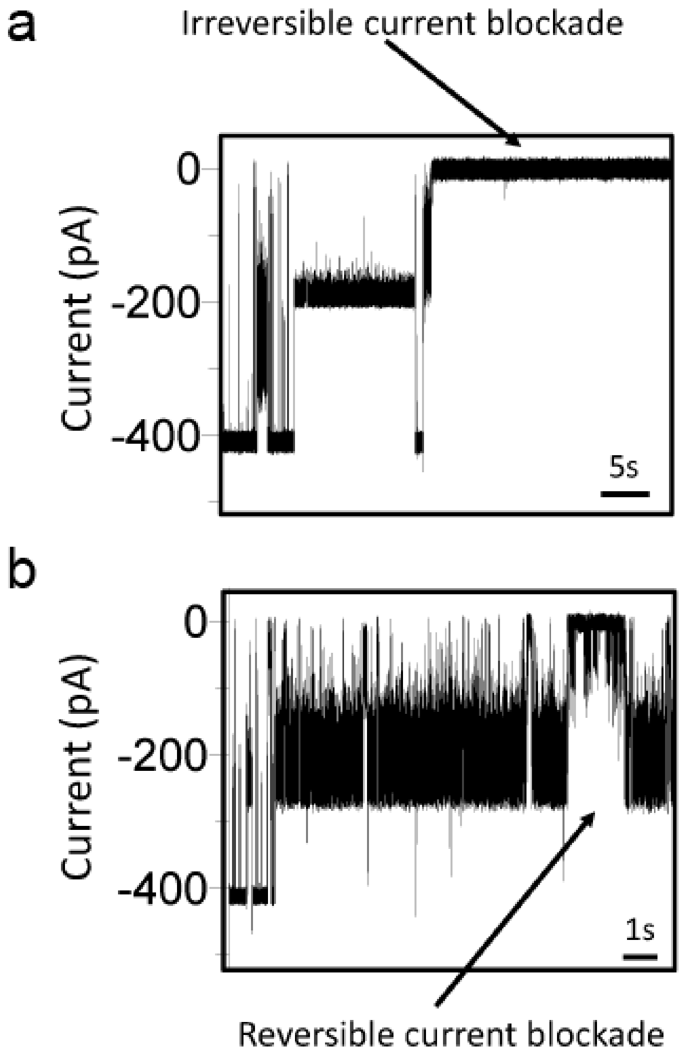

Figure S7: A representative single-channel electrical trace, acquired at a transmembrane potential of -50 mV, showing either an irreversible (**a**) or a reversible (**b**) current blockade to the fully-closed sub-state.

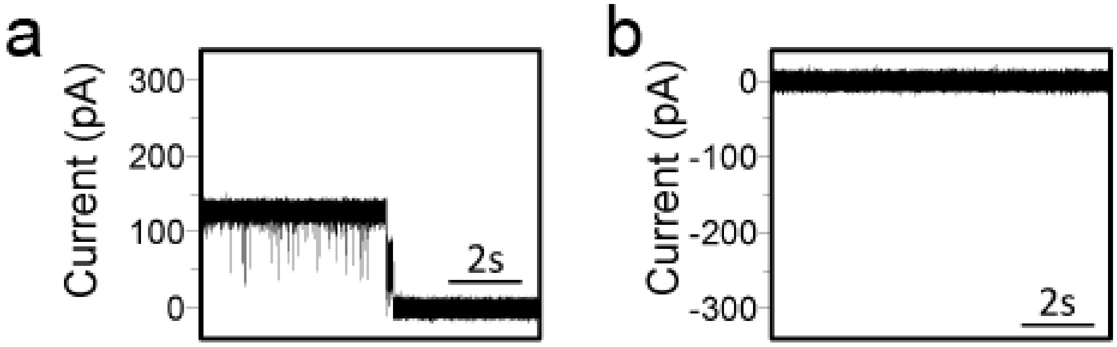

Figure S8: Subunit *d* produced greater amplitudes of the current blockades of the *c*-ring transmembrane protein pore when added at a concentration of 0.45 μM to the cytosolic side. In panels (**a**) and (**b**), the applied transmembrane potential was +30 and -30 mV, respectively. In (**a**), a long-lived current blockade to a lower conductance of ~4.1 nS was observed, which was followed by a full current blockade. At -30 mV, *c*-ring showed an open-state current decorated by frequent current blockades of varying amplitudes (**Fig. 5b**, the main text), but this was permanently and fully blocked by 0.45 μM *d* subunit added to the *cis* (cytosolic) chamber (**b**).

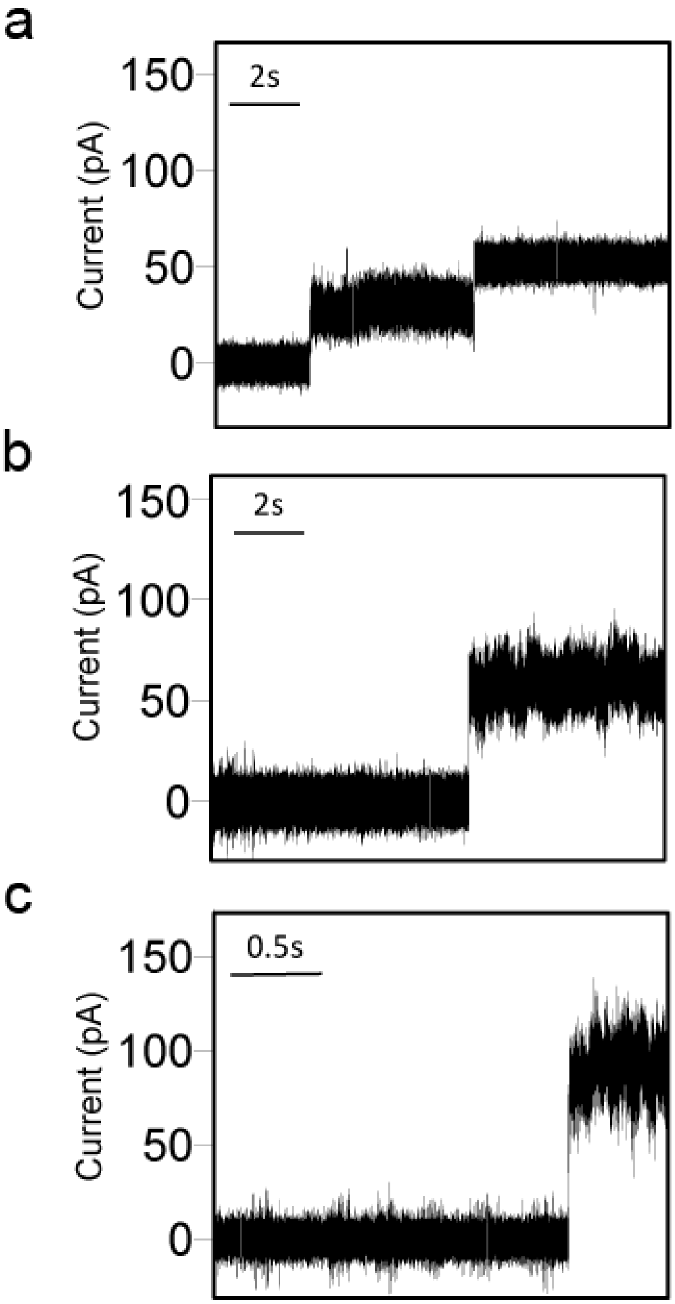

Figure S9: Some examples of single-channel insertions of the purified V_o transmembrane complex in a planar lipid membrane indicates some variability in the unitary conductance. The applied transmembrane potential was +30 mV. Amplitudes of the single-channel currents were (**a**) 32, (**b**) 60, and (**c**) 96 pA.

c AKSGVGICATCVLRPDLLFKNI
GDAGVRGSSQQPRLFVGM

c′ AKSGIGIAGIGTFKPELIMKSL
GDVGVRKYMHQPRLFVGI

c″ MNKESKDDDMSLGKF
FITGSSMIGAGVRAPRITTKNL
GATAAISDAADSALFVKI

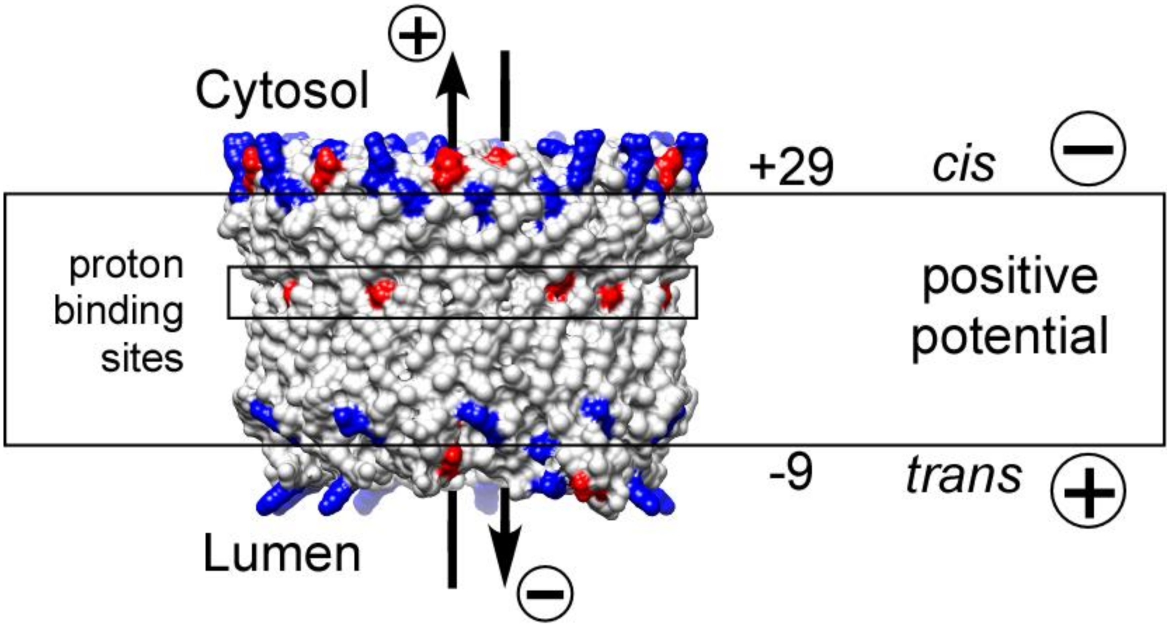

c MTELCPV
YSLGQKQ
LNSRATQDVVC-COO$^-$

c′ MSTQLASNIYAPL
GNLSPTEDY
LNTRGSE-COO$^-$

c″ YKLFTGHGSDINFGKFLLRT
SKLTVATAENMYSKS
MAGKASEFG-COO$^-$

Figure S10: Amino acid sequence of the cytosolic and vacuolar domains of the yeast V-ATPase proteolipids *c*, *c*' and *c*''. Surface representation of yeast *c*-ring. Yeast subunits *c* (Vma3p), c' (Vma11p) and c'' (Vma16p) were threaded into the crystal structure of the bacterial homolog from the *E. hirae* sodium V-ATPase (subunit K; 2bl2.pdb[7]) using the Phyre2 server[8]. Negatively and positively charged residues are highlighted in red and blue, respectively. Mass spectrometry data of intact subunits showed that the N-termini of subunit *c* (Vma3p) are acetylated and therefore carry no charge[9]. The model illustrates a clear asymmetry in the overall positive charge distribution at the cytosolic and vacuolar sites of the *c*-ring. This asymmetric charge distribution likely explains the observed uniform insertion of the *c*-ring into the planar lipid bilayer. The single orientation was conserved regardless of the polarity of the applied potential.

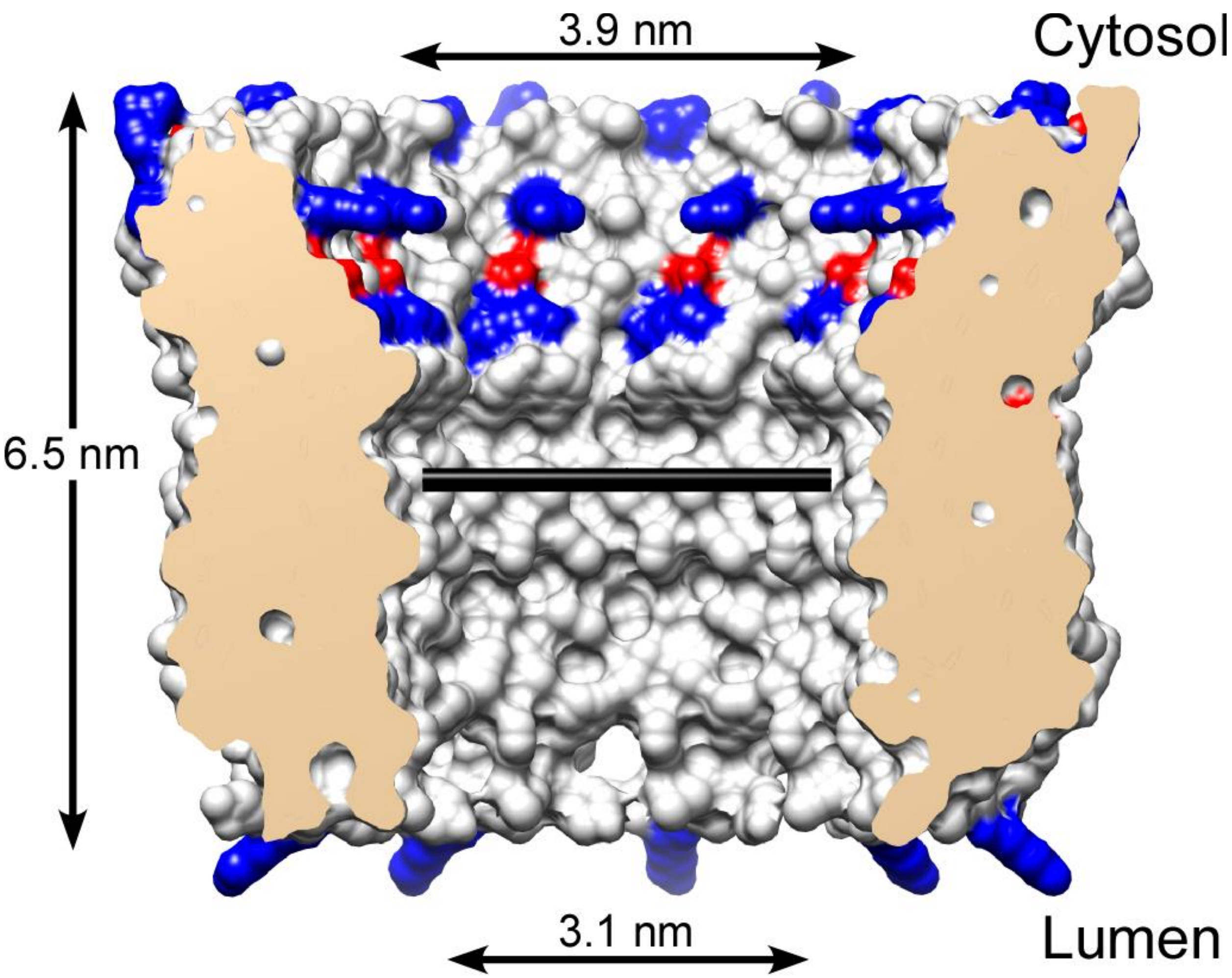

Figure S11: Internal dimensions of the yeast proteolipid *c*-ring using a molecular surface representation. This homology structure was derived using the crystal structure of the bacterial homolog from the *E. hirae* sodium V-ATPase (subunit K; 2bl2.pdb[7]) and the Phyre2 server[8]. The horizontal scale bar at the midpoint of the pore lumen measures ~3.5 nm.

References

1. Wittig, I., Braun, H.P. & Schagger, H. Blue native PAGE. *Nat Protoc* **1**, 418-28 (2006).
2. Niedzwiecki, D.J., Iyer, R., Borer, P.N. & Movileanu, L. Sampling a biomarker of the human immunodeficiency virus across a synthetic nanopore. *ACS Nano* **7**, 3341-50 (2013).
3. Cheneke, B.R., van den Berg, B. & Movileanu, L. Quasithermodynamic contributions to the fluctuations of a protein nanopore. *ACS Chem Biol* **10**, 784-94 (2015).
4. McManus, O.B., Blatz, A.L. & Magleby, K.L. Sampling, log binning, fitting, and plotting durations of open and shut intervals from single channels and the effects of noise. *Pflugers Arch* **410**, 530-53 (1987).
5. McManus, O.B. & Magleby, K.L. Kinetic states and modes of single large-conductance calcium-activated potassium channels in cultured rat skeletal muscle. *J Physiol* **402**, 79-120 (1988).
6. Movileanu, L., Cheley, S. & Bayley, H. Partitioning of individual flexible polymers into a nanoscopic protein pore. *Biophys J* **85**, 897-910 (2003).
7. Murata, T., Yamato, I., Kakinuma, Y., Leslie, A.G. & Walker, J.E. Structure of the rotor of the V-Type Na+-ATPase from Enterococcus hirae. *Science* **308**, 654-9 (2005).
8. Kelley, L.A., Mezulis, S., Yates, C.M., Wass, M.N. & Sternberg, M.J. The Phyre2 web portal for protein modeling, prediction and analysis. *Nat Protoc* **10**, 845-58 (2015).
9. Couoh-Cardel, S., Milgrom, E. & Wilkens, S. Affinity Purification and Structural Features of the Yeast Vacuolar ATPase Vo Membrane Sector. *J Biol Chem* **290**, 27959-71 (2015).
